# Basilar invagination without atlantoaxial dislocation: treatment by correction of clivus canal angle with interfacet distraction and fixation

**DOI:** 10.1186/s12891-022-06102-1

**Published:** 2022-12-29

**Authors:** Zhe Hou, Tao Fan, Wayne Fan, Qiang Jian, Yinqian Wang

**Affiliations:** 1 Department of Neurosurgery, Beijing Luhe Hospital, Capital Medica University, Beijing, People’s Republic of China; 2 Spine Center, Sanbo Brain Hospital, Capital Medical University, Beijing, People’s Republic of China; 3 Faculty of Science, The University of British Columbia, Vancouver, BC Canada

**Keywords:** Clivus canal angle, Basilar invagination, Distraction, Fixation

## Abstract

**Background:**

This study reports on the surgical technique used and clinical outcomes obtained during the treatment of basilar invagination (BI) without atlantoaxial dislocation (AAD) through the correction of the clivus canal angle (CCA) using interfacet distraction and fixation.

**Methods:**

Nineteen cases with BI without AAD treated by the correction of the clivus canal angle were retrospectively analyzed. Pre- and postoperative computed tomography scans and three-dimensional reconstruction views were obtained to measure the size of the CCA, pB-C2 distance, and degree of BI. Chiari malformation and syringomyelia were evaluated by magnetic resonance imaging (MRI). The clinical outcomes for all patients were measured using the Japanese Orthopedic Association (JOA) scale. The CCA was corrected by using interfacet distraction and fixation techniques. The Wilcoxon test was used to compare pre- and postoperative measurements.

**Results:**

All the patients were followed up for 24.95 ± 5.22 months (range 12-36 months); no patient suffered intraoperative nerve or vascular injury. Clinical symptoms improved in 17 patients (89.5%). The mean JOA score increased from 12.32 ± 1.89 to 14.37 ± 1.30 (Z = -3.655, *P* < 0.001). The mean CCA improved from 129.34 ± 8.52° preoperatively to 139.75 ± 8.86° postoperatively (Z = -3.824, *P* < 0.001). The mean pB-C2 decreased from 7.47 ± 2.21 to 5.68 ± 3.13 (Z = -3.060, *P* = 0.002). Syringomyelia was significantly reduced in 10 out of 13 patients by the first follow-up year. All patients achieved bony fusion.

**Conclusion:**

Posterior interfacet distraction and fixation to correct the CCA is a feasible and effective method for treating BI without AAD.

## Introduction

Basilar invagination (BI) is a condition in which the odontoid process protrudes upwards into the skull base, causing the compression of the structures in that area, leading to neurological damage [1]. In 2004, Goel classified BI into A and B based on whether there was a combined atlantoaxial dislocation (AAD) or not [2]. Group A can now be effectively treated by the reduction and fixation of the AAD. However, the treatment strategy for Group B, not complicated by AAD, is ambiguous and without consensus. Very few studies have examined the surgical treatment strategies for Group B [3, 4]. The commonly used surgical methods for treating BI without AAD include anterior transoral dentate resection, posterior cranial fossa decompression, and atlantoaxial fixation, but there is no consensus on the appropriate surgical approach and method [2, 5–7].

Studies on AAD found that reducing the clivus canal angle (CCA) by adjusting the position of the odontoid process relieves the pressure on the ventral side of the brainstem [8]. Our previous study, where we treated BI complicated by AAD using inter-articular release and fixation, yielded good results [9]. Considering that the CCA is usually reduced in patients with AAD, we have tried to use a technique that corrected the CCA to treat this group of patients since 2018. The objective of this article is to report the early results of the treatment of BI without AAD by correcting the CCA.

### Materials and methods

#### Patient population

From May 2019 to April 2021, patients with BI without AAD were treated with interfacet distraction and fixation to correct the CCA. All the procedures were performed by the same surgeon. The hospital’s ethics committee board approved this study. All patients gave informed consent.

The diagnosis of BI was made when the tip of the odontoid process was more than 5 mm above the Chamberlain’s line. Fusion of the first cervical vertebrae (C1) with any part of the occipital bone was defined as the occipitalization of C1. Patients were excluded if they had AAD, history of trauma, prior occipitocervical surgery, or infection.

The clinical features are summarized in Table 1. Sensory dysfunction was the main complaint in 15 patients (78.9%), motor dysfunction in 10 patients (52.6%), occipitocervical pain in five patients (26.3%), and ataxia in eight patients (42.11%). The clinical status before and at least one year after surgery was assessed using the Japanese Orthopedic Association (JOA) scores (Table 2).

**Table 1:** Clinical features in 19 patients

Variable	All patients (*n* = 19)
Demographics	
Mean age, years(range)	41.8 ± 12.1(20–66)
Sex(M/F)	6:13
Clinical symptoms, n (%)	
Sensory disturbance	15 (78.9%)
Motor dysfunction	10(52.6%)
Neck pain	5 (26.3%)
Ataxia	8 (42.11%)
Radiologic features, n (%)	
Occipitalization of C1	8 (42.1%)
Chiari malformation	100 (100%)
Syringomyelia	13 (68.4%)

**Table 2:** Summary of radiologic and clinical date obtained in 19 patients

Patient no.	Sex/ Age (year)	CL (mm)	complication	CCA, (deg)		pB-C2 (mm)		JOA		Follow-up (month)	fusion
				Pre	Post	Pre	Post	Pre	Post		
1	F/35	17.8	None	126.8	139.5	6.8	4.7	12	15	36	+
2	F/66	11.8	None	130.6	137.5	6.5	4.4	11	15	26	+
3	F/33	9.6	None	134.5	147.5	4.5	2.1	13	15	24	+
4	F/31	10.6	None	126.4	137.5	8.5	5.8	12	15	24	+
5	F/27	8.1	None	130.2	139.7	6.3	3.7	9	12	24	+
6	F/30	14.1	None	127.1	138.3	3.8	1,5	9	11	24	+
7	M/61	12.4	None	135.9	146.7	7.9	6.8	11	14	30	+
8	F/40	7.7	None	143.1	152.4	8.1	7.6	13	15	30	+
9	M/51	6.2	None	128.3	135.6	4.5	1.9	13	16	18	+
10	F/40	7.8	None	137.3	148.5	7.3	3.2	14	14	30	+
11	M/56	12.1	None	119.4	128.7	4.9	2.1	12	14	24	+
12	M/28	15.4	CSF leak	109.3	121.3	10.3	6.6	15	16	24	+
13	F/59	11.2	None	129.4	143.6	11.3	8.2	13	14	24	+
14	F/33	15.6	None	132.9	141.6	9.1	6.1	9	13	28	+
15	F/37	5.9	None	136.7	146.7	8.9	7.6	12	14	28	+
16	F/32	19.8	None	111.9	119.4	9.6	6.3	13	14	26	+
17	F/40	5.9	None	134.7	143.1	7.7	4.4	15	16	24	+
18	M/51	12.3	Delayed healing of incision	136.5	148.9	10.6	8.8	15	15	18	+
19	M/44	11.7	None	126.5	138.8	5.4	2.6	13	15	12	+

#### Radiological studies

Pre and postoperative thin-slice computed tomography (CT) scans and magnetic resonance imaging (MRI) were obtained for all patients. The measurements were all performed independently by two neurosurgery residents, and the measurements were averaged. CCA and pB-C2 were used to evaluate the degree of ventral brainstem compression. At the most recent follow-up, these parameters were analyzed and compared with the preoperative data.

CT scans were performed three to six months after surgery to check the position of the bone mass or cage and whether bone fusion had occurred between the lateral block joints. We performed another examination after six months to confirm if bone fusion had occurred.

#### Surgical procedure

Interfacet manipulation and distraction technique were used to treat BI without AAD in all patients.

**Fig. 1:**
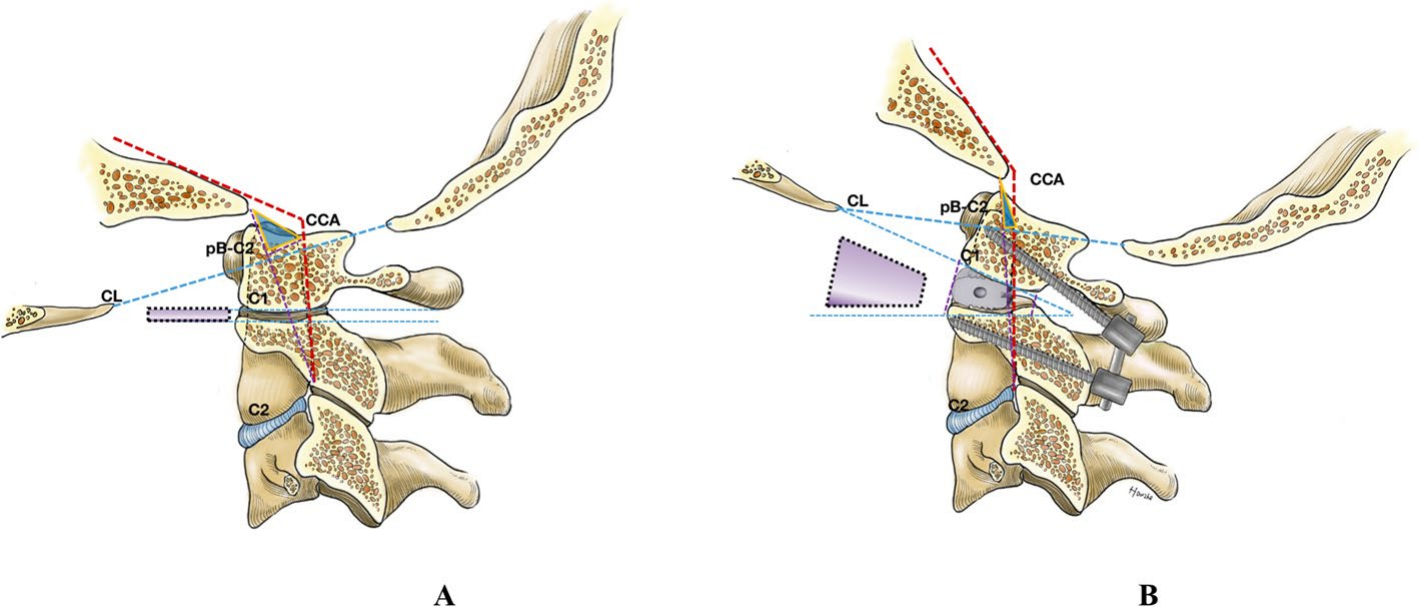
Diagram showing the correction of the CCA with interfacet distraction and fixation. **A** The clivus-canal angle (CCA) is the angle between the plane of the clivus and the posterior edge of the axial vertebrae. The CCA tends to be smaller in basilar invagination (BI) without atlantoaxial dislocation (AAD). The articular surface of BI without AAD tends to be parallel or only slightly tilted anteriorly and posteriorly in the sagittal position. **B** The lateral atlantoaxial articulation consists of the inferior C1 articular surface and the superior C2 articular surface. After the articular surface was released, bone blocks were inserted to assist in the correction of the CCA. It is equivalent to the descent of the odontoid process and the upward and backward rotation of C1. For atlantoaxial fixation, cantilever technology can be used to assist in increasing the CCA. After fixation and increase of the CCA, a trapezoid was formed between the upper articular surfaces in the sagittal position, with its lower base in front. The blue triangle area decreased after the operation, suggesting that the compression on the ventral side was reduced

All operations were performed under intraoperative neuro-physiological monitoring. After general anesthesia, each patient was placed in the prone position with the head secured in a Mayfield head-holder device and a neutral position. A suboccipital posterior median approach that exposed from the posterior occipital bone to the C2 spinous process was used. The C2 ganglion was retracted upward under the microscope to allow for interarticular manipulation. The articular cartilages of the upper and lower articular surfaces were removed by an ultrasonic osteotome under microscopic guidance. Cortical bone blocks or autogenous bone fragments were taken from the resected C1 posterior arch and occipital bone. Interfacet manipulation and distraction were performed using special distraction instruments as we have described earlier [9]. Two cortical bone blocks or cages of approximately 6 mm in height filled with bone debris were implanted into the intra-articular spaces bilaterally. The bone blocks or cages were placed on the ventral side of the articular surface. C1 lateral mass screws and C2 pedicle screws were placed using the Goel technique, and occipital-C2 screws were placed in some cases of occipitalization of C1. The head frame was adjusted so that the head position was slightly overextended to facilitate the improvement of the CCA. The titanium rods, shaped to the appropriate length and shape, were alternately repositioned using the cantilever technique. Finally, the screws were tightened. At this point, the C2 vertebra leaned forward and the CCA increased (Fig. 1). C-Arm X-ray images were obtained before interarticular manipulation and after internal fixation to determine the position of the cages and ensure that the CCA was corrected. Generally, the odontoid process will move down slightly, and the CCA will increase by 10–15 degrees.

### Statistical analysis

Statistical analyses were performed using the SPSS 22.0 software. All parameters were expressed as mean ± standard deviation. The pre- and postoperative parameters were analyzed using the Wilcoxon test. The significant difference was set at *P* < 0.05 (Table 3).

**Table 3:** Statistical outcomes in 19 patients

Variable	Pre-mean Value^a^	Post-mean value		Two-tailed Wilcoxon signed-rank test
		2 weeks^b^	the latest^c^	
CCA°	129.34 ± 8.52°	139.75 ± 8.86°	139.75 ± 8.86°	< 0.001
pB-C2 (mm)	7.47 ± 2.21	5.68 ± 3.13	5.68 ± 3.13	= 0.002
JOA	12.32 ± 1.89	14.37 ± 1.30	14.58 ± 1.26	< 0.001

*JOA *Japanese Orthopedic Association scores, *CCA* Clivus-canal angle, pB-C2, The length of a perpendicular line extending from another line drawn from the basion to the dorsal-caudal aspect of the C2 body on sagittal CT images

^a,b,c^The mean value of CCA, pB-C2, and JOA at different times

## Results

### Operative results

In 12 patients, C1-C2 screws were placed. The other five cases of occipitalization of C1 and two cases of atlas dysplasia were considered unsuitable for C1 screw placement, and occipital-C2 screws were placed instead. In this group, cages were placed in 17 cases (34 sides) and bone blocks (4 sides) were placed in 2 cases. The mean operative time was 118.4 min (90–155 min), and the mean intraoperative blood loss was 121.1 mL (85–200 mL). No infections or deterioration of neurological function occurred postoperatively. The mean JOA score increased in 17 patients by the first follow-up year, and two patients had stable symptoms. The average JOA score was 12.32 ± 1.89 preoperatively and 14.37 ± 1.30 (Z = -3.655, *P* < 0.001) at the latest follow-up. The cages or bone blocks were placed between the articular surfaces of the C1-C2 lateral blocks in all patients. One case had a unilateral collapse of the C1 lower articular surface without nerve or vascular damage. All 19 patients achieved inter-articular bony fusion by the last follow-up visit.

### Radiographic results

**Fig. 2:**
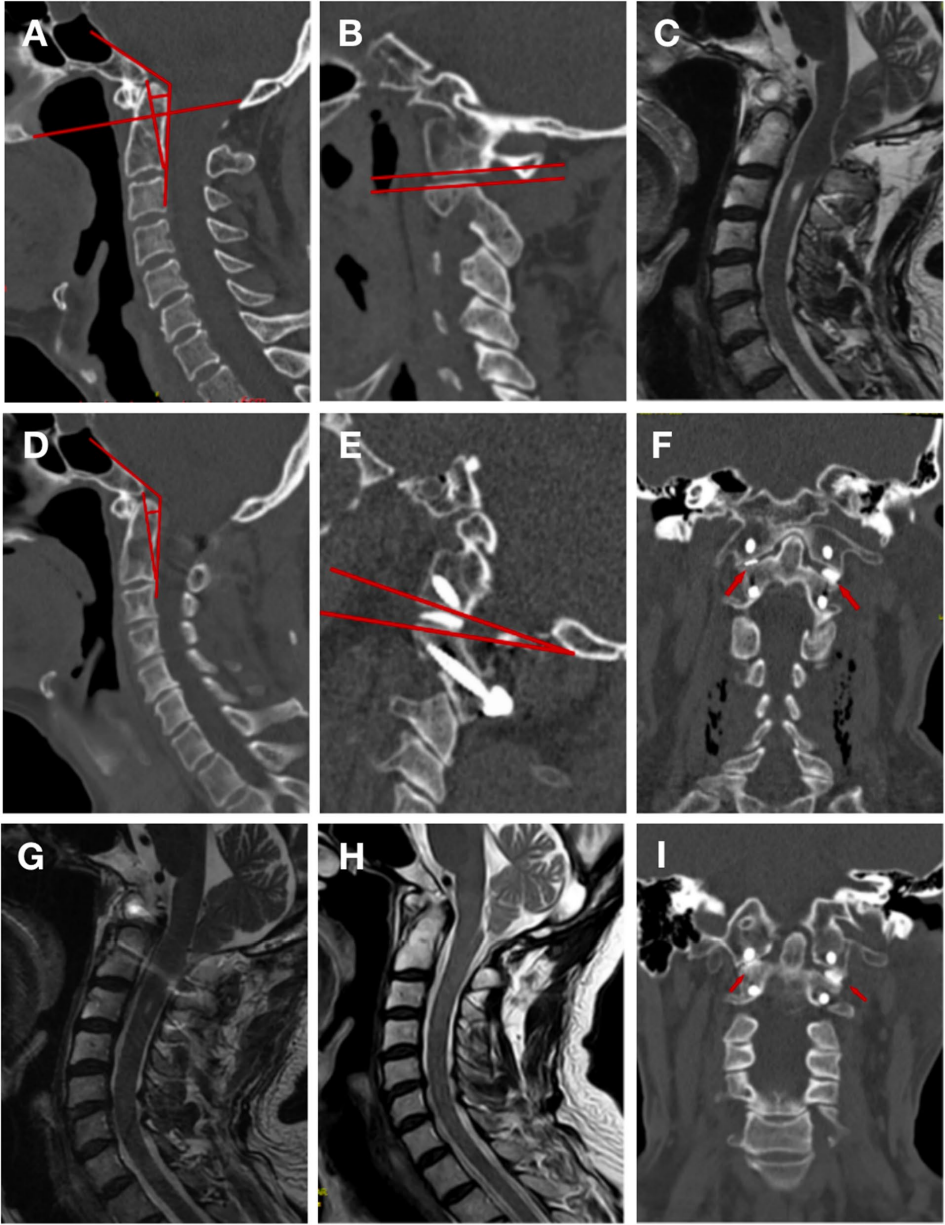
A 51-year-old man presented with gait disturbance and dizziness for six months. The symptoms of the patient significantly improved postoperatively, and the JOA score increased from 13 before the operation to 16. **A** and **B**: Sagittal reconstructed computed tomography (CT) scan showing the presence of occipitalization of C1, basilar invagination (BI) without atlantoaxial dislocation (AAD), clivus-canal angle (CCA) = 128.3°, pB-C2 = 5.3 mm, and that the relationship of the two facets of the C1–C2 joint was relatively parallel (red lines). **C**: Preoperative magnetic resonance imaging (MRI) scan showing associated Chiari malformation and syringomyelia. **D**: Postoperative sagittal CT showed that the odontoid process decreased slightly and the CCA increased; CCA = 135.6° and pB-C2 = 2.7 mm. The two facets of the C1–C2 joint are not parallel **E**. **F**: The facets of C1 and C2 were released, and an autologous bone block was implanted. **G**: Sagittal MRI showed a slight decrease in the syringomyelia and a slight upward movement of the tonsillar herniation. The 30-month follow-up showed that the syringomyelia disappeared completely and the tonsillar herniation was reduced (**H**), and bony fusion occurred as confirmed on the CT scan **I**

**Fig. 3:**
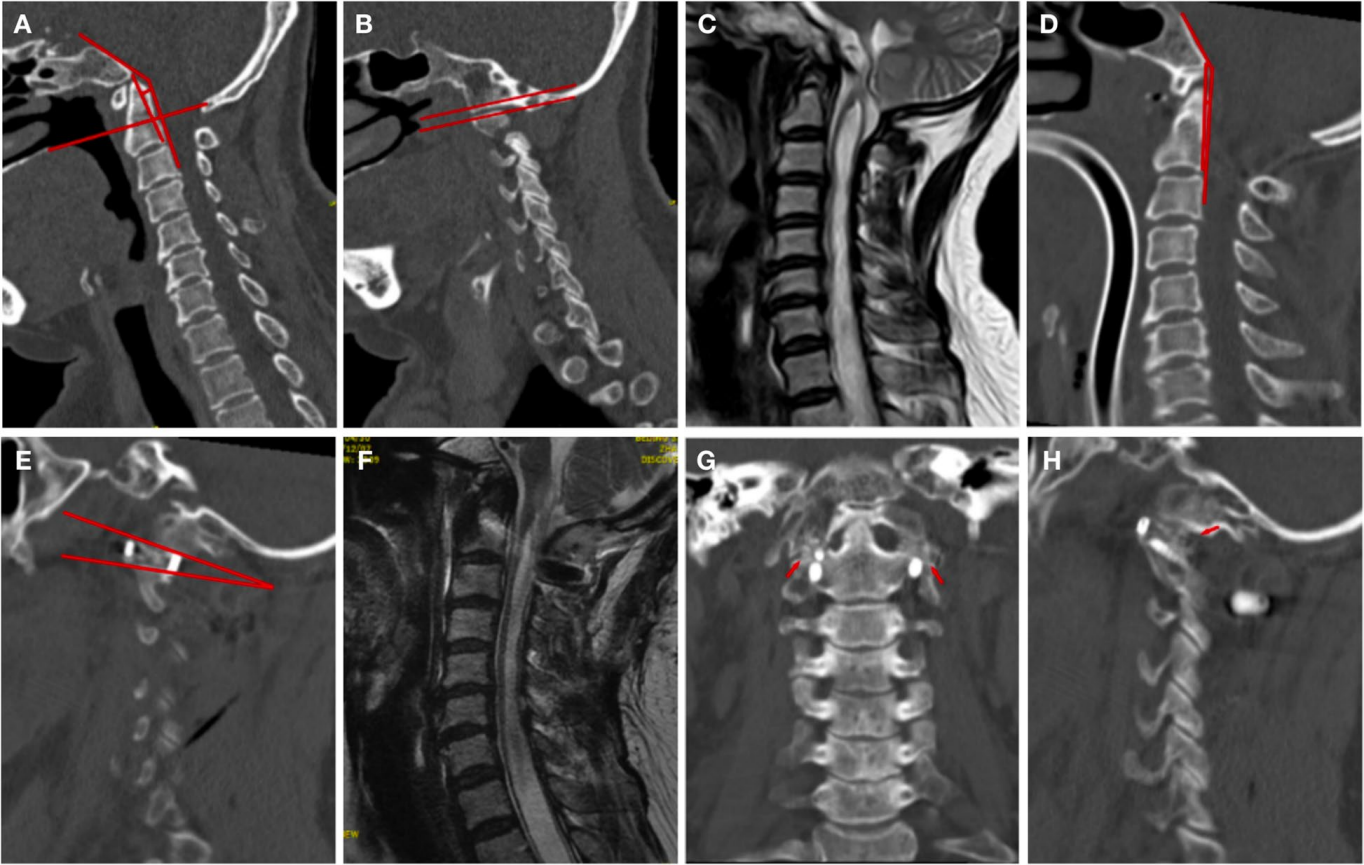
A 40-year-old woman presented with sensory disturbance, neck pain, and right-hand muscle atrophy for 15 months. After the operation, the patient’s sensory disturbance and neck pain were significantly relieved, and the Japanese Orthopedic Association (JOA) score increased from 13 to 15. **A**: Sagittal reconstructed computed tomography (CT) scan shows the presence of occipitalization of C1, BI without atlantoaxial dislocation (AAD), clivus-canal angle (CCA) = 143.1°, pB-C2 = 4.5 mm, and that the two facets of the C1–C2 joint were parallel (red lines) **B**. **C**: Preoperative magnetic resonance imaging (MRI) scan showing associated Chiari malformation, and syringomyelia. **D**: Postoperative sagittal CT showed that the odontoid process was lowered and the CCA increased, CCA = 152.4° and pB-C2 = 1.9 mm. The two facets of the C1–C2 joint are not parallel; the C1 joint rotates upward and backward **E**. **F**: Postoperative sagittal MRI showed a slight decrease in syringomyelia. **G** and **H**: The 12-month CT showed that bony fusion had occurred between the C1–C2 articular surfaces

**Fig. 4:**
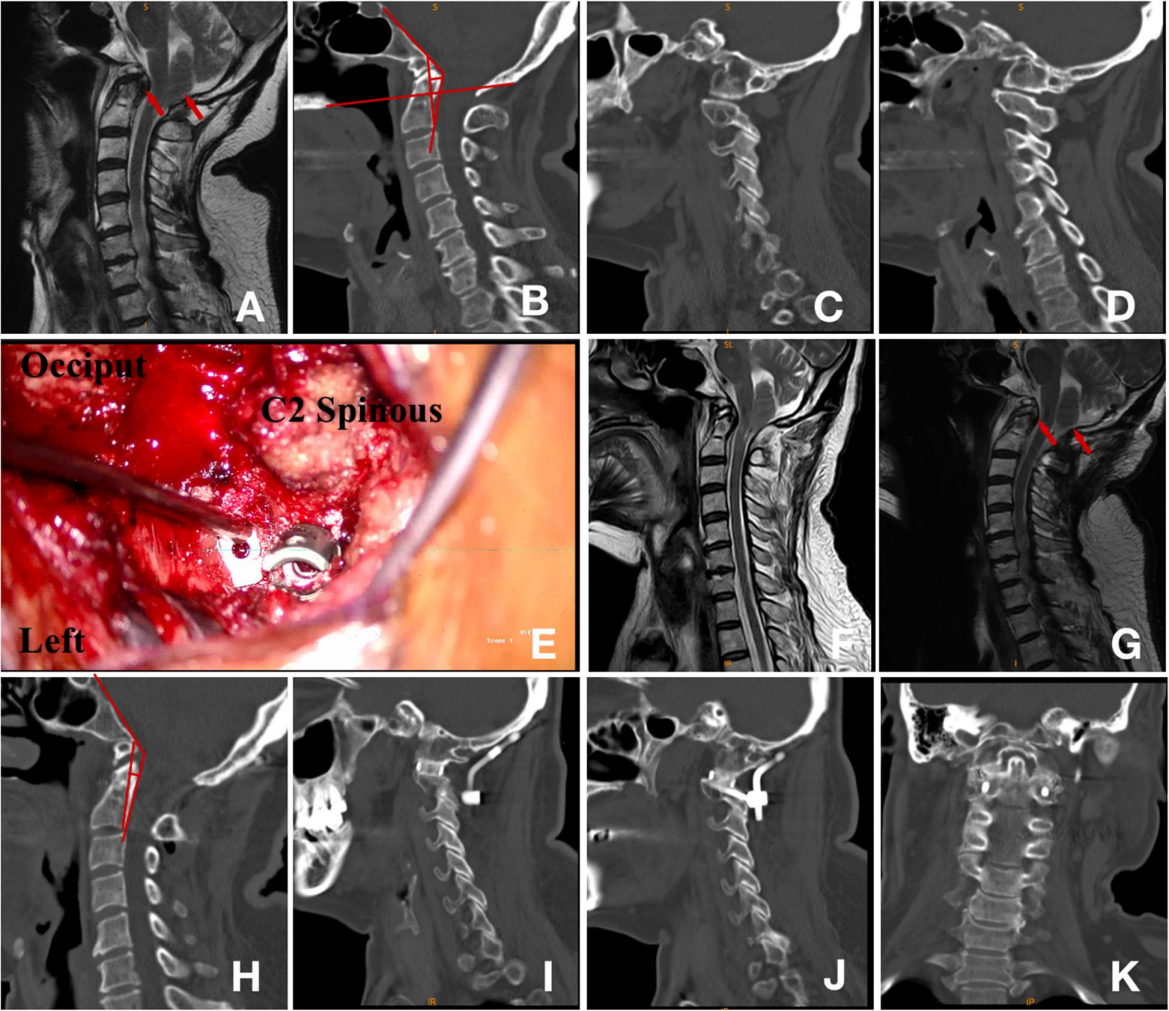
A 66-year-old woman presented with sensory disturbance and unsteady walking for more than 4 years. After the operation, the patient’s unsteady walking was significantly relieved, and the Japanese Orthopedic Association (JOA) score increased from 11 to 15. **A**: Preoperative sagittal magnetic resonance imaging (MRI) showed tonsillar hernia, large soft tissue volume behind the odontoid process, and ventral compression of the brain stem. **B**: Preoperative sagittal computed tomography (CT) showed basilar invagination (BI), occipitalization of C1, clivus-canal angle (CCA) = 130.6°, pB-C2 = 6.5 mm, and no deformity of the bilateral lateral atlantoaxial joint **C**, **D**. **E**: During the operation, the cage was placed between the joints and then screws were placed. **F**: Sagittal MRI on the third day after operation showed that the compression on the ventral side of the brain stem was reduced compared with that before operation, and the descending degree of the tonsillar hernia had no significant change compared with that before operation. **G**: Three months after operation, MRI showed that the descending degree of tonsillar hernia was significantly reduced, the volume of soft tissue behind the odontoid process was smaller than that before operation, and the ventral compression of brain stem was relieved. **H**: Six months after operation, CT scan showed increased CCA, CCA = 137.5°, pB-C2 = 4.4 mm, and bone fusion can be seen between the joints of the bilateral lateral atlantoaxial joint **I**, **J**, **K**

Table 1 shows that Chiari malformations were found in 19 patients (100%), and syringomyelia was demonstrated in 13 patients (68.4%). Correction of CCA was achieved in all 19 patients; this was confirmed on postoperative CT scans (Figs. 2, 3 and 4). The mean CCA improved from 129.34 ± 8.52° preoperatively to 139.75 ± 8.86° postoperatively (Z = -3.824, *P* < 0.001). The pB-C2 went from 7.47 ± 2.21 mm preoperatively to 5.68 ± 3.13 mm postoperatively (Z = -3.060, *P* = 0.002). A reduction of the syrinx’s size was observed in 10 patients (85.4%) one year postoperatively, with no change in syringomyelia occurring during follow-up in three cases. The correction was maintained in all patients at follow-up.

### Complications

Two patients had short-term complications: One had delayed wound healing and the other had a cerebrospinal fluid leak treated with lumbar pool drainage. Complications are summarized in Table 2.

## Discussion

BI without AAD is now considered to be a stable type with no evidence of atlantoaxial instability on dynamic X-ray and CT scans. The literature review showed no definitive treatment strategy. The common surgical approach is posterior fossa decompression, including bony and subdural decompression; however, many patients with BI, treated by this procedure, fail to show improvement [10, 11]. Anterior decompression is still considered the classic surgical method; however, anterior oral surgery is complex, has many complications, and additionally requires posterior fixation to maintain stability [12, 13]. Goel et al. believed that the patients’ symptoms were the result of atlantoaxial instability; he achieved good results with direct posterior fixation [14]. Although posterior fixation alone has shown better results, it is not effective in all cases, and instances of re-aggravation have been reported [15].

Ventral brainstem compression is a common pathogenic feature, the evaluation of which is crucial in the treatment of BI. pB-C2 is an important evaluation index that many researchers have used to assess the degree of ventral brainstem compression in BI [13, 15]. In our case, pB-C2 decreased from a mean of 7.47 ± 2.21 mm preoperatively to 5.68 ± 3.13 mm postoperatively, suggesting that direct posterior surgery in patients with BI without AAD can reduce ventral brainstem compression. The CCA has been used in many studies to measure the degree of the ventral cervical spinal cord compression at the craniovertebral junction, which is a very important indicator of the reduction of AAD [8, 16]. The CCA at a neutral position in normal people measured by some scholars was 145-160° [17]. Botelho and Ferreira measured the CCA in 25 patients with BI, and the average angle in this group was 120° (79–145°) [18]. There are no targeted studies in cases of BI without AAD, and in this group, the CCA tends to be significantly reduced compared to normal.

Goel et al. suggest that the shortening of the neck and height of the cranial fossa is not related to the shortening of the length of the brainstem and spinal cord. The former may be a naturally occurring protective measure that facilitates the inelastic traversal of the spinal cord at the tip of the dentate [3]. Owing to these compensatory mechanisms, the ventral compression of the brainstem in this group of cases is often not serious. Appropriate ventral decompression may improve symptoms. Previous studies that used biomechanical principles similar to those of our study to improve the CCA in the treatment of BI showed promising results in some cases [4, 19]. Henderson’s finite element analysis shows that the normalization of CCA and the stability of fusion are related to clinical improvement [20].

The correction of the CCA is very important. We have found that if internal fixation leads to a reduction of the CCA, in some cases, it will aggravate the ventral compression of the brainstem, resulting in no improvement or even aggravation of symptoms. This phenomenon is explained in the literature by Tachibana et al. [21], who found that cervical movements were important in the formation of spinal cavities. They performed the Queckenstedt test on patients with Chiari malformation combined with syringomyelia and found that none had obstruction of cerebrospinal fluid flow when the neck was extended or in a neutral position, and all had obstruction of cerebrospinal fluid flow when the neck was flexed. Wang et al. suggest that a satisfactory reduction can be achieved by repositioning the CCA by 150° or more [8]. Nagashima and Kubota believed that if the CCA was less than 130°, there might be ventral compression of the brainstem, and it should be corrected to a greater angle during surgery [22]. Botelho’s case report suggests that enlarging the CCA in patients with BI can reduce the compression of the ventral brainstem [10].

Given this situation, we have improved the interfacet distraction and fixation technique to allow for ventral decompression and preservation of atlantoaxial stability through a one-stage procedure. We have attempted to treat BI by expanding the CCA with internal fixation, thereby reducing the ventral compression of the brainstem. In our group of 19 patients, symptoms improved after posterior surgery was performed to increase the CCA. Intraoperatively, we performed microsurgical interfacet release and placed a bone block or cage of approximately 6 mm in height filled with bone debris ventral to the articular surface. The head frame was subsequently adjusted to place the head in a slightly hyperextended position, which is equivalent to a rotational movement of C1 using the bone block or cage as a fulcrum, increasing the CCA, before fixation was applied. The improvement in ventral compression is because of two factors. Firstly, the odontoid process drops to a lower level relative to the C1 vertebra after the interarticular placement of the bone block or cage. This directly reduces the ventral compression. Secondly, with C1 and clivus tilted slightly upwards and backwards after fixation, the inferior articular surface of C1 is not parallel to the superior articular surface of the axis but is rather trapezoidal in the sagittal position (Fig. 1). Therefore, the CCA is increased, which in turn reduces the ventral compression.

The advantage of this technique is that ventral decompression and stabilization of C1 and C2 can be achieved through a posterior one-stage procedure, avoiding the more invasive transoral release followed by posterior fixation. Although this technique requires interarticular manipulation of the lateral atlantoaxial articulation, it tends to be horizontal or only slightly tilted anteriorly and posteriorly in this type of case, unlike in cases of AAD where the articulation tends to be severely anteriorly tilted, and thus the intraoperative interarticular release and manipulation are not complicated.

Some details need attention during the operation: Firstly, the position of the bone block or cage is very important, as it should be placed on the anterior side of the articular surface to facilitate a slight posterior rotation of the atlas. Secondly, the adjustment of the head frame is also noteworthy, as the lateral articulation is deeper in patients with BI, and sometimes the head needs to be adjusted to a slightly flexed position to facilitate exposure, with subsequent slight hyperextension before tightening of the screws after fixation. If necessary, cantilever technology can be used to assist in increasing the CCA. Thirdly, the inter-articular release should be done with gentle and gradual extension. It is often difficult in patients with BI without AAD to hold the lateral block joint open for a greater distance after release, and it is significantly more difficult to place the cage between joints when the cage height exceeds 6 mm, even if the articular surfaces have been treated with an ultrasonic osteotome. In this group, the placement of a cage in the interarticular space on one side resulted in a fracture of the inferior C1 articular surface, but fortunately, there was no resultant neurological or vascular damage.

BI often occurs with syringomyelia [23], as was seen in 13 cases in our study. Recent studies suggest that syringomyelia is secondary to atlantoaxial instability, a view that is confirmed in many cases of BI [3]. The technique of improving ventral compression through surgical fixation is effective. In our group of 13 cases with syringomyelia, improvement of the CCA without posterior fossa and subdural decompression was achieved, and there was significant relief of syringomyelia in 10 cases at the postoperative follow-up. The improvement of syringomyelia may be because of the relief of the ventral brainstem compression, which subsequently improves the cerebrospinal fluid flow patterns at the craniovertebral junction [24]. The absence of relief of the cavity in individual cases may be explained by insufficient ventral decompression.

There were no serious complications in this group, and most of the patients’ symptoms were significantly relieved.

The limitations of this study need to be noted. As this is a retrospective analysis of a series by the same surgeon, there may be an inherent bias in case selection and treatment. Patients were only followed up for 2.1 years, and the long-term outcomes and complications need to be observed further. The increase in the CCA of type B BI can reduce the compression of the ventral brainstem, but the best degree of CCA correction in such cases still needs further study. Furthermore, in some patients with combined severe platybasia, exposure of the lateral atlantoaxial joint via the posterior approach is difficult and this procedure is inappropriate in such cases.

## Conclusion

The treatment of BI without combined AAD is challenging, and this study provides a strategy to resolve ventral compression in such cases by correcting the CCA in a one-stage posterior approach. A modified posterior fixation approach with posterior inter-articular release and fixation can effectively correct the CCA. This technology is feasible, safe, and effective.

## Data Availability

All data generated or analyzed during this study are included in this published article.

## References

[1] Goel A (2009). Basilar invagination, Chiari malformation, syringomyelia: a review. Neurol India.

[2] Goel A (2004). Treatment of basilar invagination by atlantoaxial joint distraction and direct lateral mass fixation. J Neurosurg Spine.

[3] Goel A, Nadkarni T, Shah A, Sathe P, Patil M (2016). Radiologic evaluation of basilar invagination without obvious atlantoaxial instability (Group B Basilar Invagination): analysis based on a study of 75 patients. World Neurosurg.

[4] Klekamp J (2014). Treatment of basilar invagination. Eur Spine J.

[5] Menezes AH (1991). Anterior approaches to the craniocervical junction. Clin Neurosurg.

[6] Menezes AH. Surgical approaches: postoperative care and complications “transoral-transpalatopharyngeal approach to the craniocervical junction”. Childs Nerv Syst. 2008;24(10):1187–93. 10.1007/s00381-008-0599-3 18389262

[7] Vidal CHF, Brainer-Lima AM, Valenca MM, Farias RL (2019). Chiari 1 malformation surgery: comparing non-violation of the arachnoid versus arachnoid opening and thermocoagulation of the Tonsils. World Neurosurg.

[8] Wang C, Yan M, Zhou HT, Wang SL, Dang GT (2006). Open reduction of irreducible atlantoaxial dislocation by transoral anterior atlantoaxial release and posterior internal fixation. Spine (Phila Pa 1976).

[9] Shang G, Fan T, Hou Z (2019). A modified microsurgical interfacet release and direct distraction technique for management of congenital atlantoaxial dislocation: technical note. Neurosurg Rev.

[10] Botelho RV, Neto EB, Patriota GC, Daniel JW, Dumont PA, Rotta JM (2007). Basilar invagination: craniocervical instability treated with cervical traction and occipitocervical fixation. Case report. J Neurosurg Spine.

[11] Grabb PA, Mapstone TB, Oakes WJ (1999). Ventral brain stem compression in pediatric and young adult patients with Chiari I malformations. Neurosurg.

[12] Shriver MF, Kshettry VR, Sindwani R, Woodard T, Benzel EC, Recinos PF (2016). Transoral and transnasal odontoidectomy complications: a systematic review and meta-analysis. Clin Neurol Neurosurg.

[13] Zileli M, Cagli S (2002). Combined anterior and posterior approach for managing basilar invagination associated with type I Chiari malformation. J Spinal Disord Tech.

[14] Goel A, Sathe P, Shah A (2017). Atlantoaxial fixation for basilar invagination without obvious atlantoaxial instability (Group B Basilar Invagination): outcome analysis of 63 surgically treated cases. World Neurosurg.

[15] Salunke P, Karthigeyan M, Malik P, Panchal C. Changing perception but unaltered reality: how effective is C1-C2 fixation for Chiari malformations without instability? World Neurosurg. 2020;136:e234–44. 10.1016/j.wneu.2019.12.122 31899405

[16] Chen Z, Duan W, Chou D (2021). A safe and effective posterior intra-articular distraction technique to treat congenital atlantoaxial dislocation associated with Basilar Invagination: case series and technical nuances. Oper Neurosurg (Hagerstown).

[17] Henderson FC, Sr., Henderson FC, WAt W, Mark AS, Koby M (2018). Utility of the clivo-axial angle in assessing brainstem deformity: pilot study and literature review. Neurosurg Rev.

[18] Botelho RV, Ferreira ED (2013). Angular craniometry in craniocervical junction malformation. Neurosurg Rev.

[19] Kim LJ, Rekate HL, Klopfenstein JD, Sonntag VK (2004). Treatment of basilar invagination associated with Chiari I malformations in the pediatric population: cervical reduction and posterior occipitocervical fusion. J Neurosurg.

[20] Henderson FC, Wilson WA, Mott S (2010). Deformative stress associated with an abnormal clivo-axial angle: a finite element analysis. Surg Neurol Int.

[21] Tachibana S, Iida H, Yada K (1992). Significance of positive queckenstedt test in patients with syringomyelia associated with Arnold-Chiari malformations. J Neurosurg.

[22] Nagashima C, Kubota S (1983). Craniocervical abnormalities. Modern diagnosis and a comprehensive surgical approach. Neurosurg Rev.

[23] Collignon FP, Cohen-Gadol AA, Krauss WE (2004). Circumferential decompression of the foramen magnum for the treatment of syringomyelia associated with basilar invagination. Neurosurg Rev.

[24] Fan T, Zhao H, Zhao X, Liang C, Wang Y, Gai Q (2017). Surgical management of Chiari I malformation based on different cerebrospinal fluid flow patterns at the cranial-vertebral junction. Neurosurg Rev.

